# The Health Economics Section: scope and future

**DOI:** 10.1093/heapol/czag090

**Published:** 2026-09-18

**Authors:** Peter Binyaruka, Neha Batura, Beverley M Essue

**Affiliations:** Department of Health System, Impact Evaluation and Policy, Ifakara Health Institute, PO Box 78373, Dar es Salaam, Tanzania; Global Business School for Health, University College London, Marshgate Building, 7 Sidings St., London E20 2AE, United Kingdom; Institute of Health Policy, Management and Evaluation, University of Toronto, 155 College Street W, Toronto, ON, Canada M5T 1P8

## Introduction: health economics in a global context

Health economics has never been more central to the questions confronting health systems and policymakers. Across low- and middle-income settings in particular, governments are being asked to do more with constrained resources, respond to demographic and epidemiological transitions, integrate new technologies, and confront persistent, and in many cases widening inequities in access, quality, and financial protection. These challenges have sharpened demand for economic evidence that does not merely describe costs but helps decision-makers understand trade-offs, set priorities, and design policies that improve system performance over time. *Health Policy and Planning* has long served as a forum for this type of work.

The Health Economics Section at *Health Policy and Planning* is explicitly oriented toward scholarship that engages with real-world policy and system challenges through a rigorous economic lens. Health economics occupies a distinctive and important place within the journal. The section is grounded in the view that economics is not merely a technical exercise, but that applied health economics research can inform and ground understanding of how health systems allocate resources, respond to incentives, and translate resource allocation decisions into health and social outcomes.

This section is intended for readers working at the interface of analysis and action: health economists engaged in applied policy research; health systems and policy scholars drawing on economic methods and theories; and decision-makers in ministries of health and finance, development agencies, and health system organizations. The common thread across this readership is a need for evidence that clarifies trade-offs, informs prioritization, and can support action to improve the efficiency, equity, and sustainability of health systems. Papers in this section speak directly to these concerns, situating empirical findings within the institutional and policy contexts in which decisions are made.

This editorial reflects on the evolution of the Health Economics Section in *Health Policy and Planning* over the past 5 years by examining the major themes, publication patterns, and geographical distribution of published articles. It also identifies emerging priorities, underexplored areas, and highlights potential areas for future health economics research. In doing so, the editorial aims to stimulate broader scholarly engagement and encourage submissions that address the evolving financing, equity, and sustainability challenges facing health systems globally, particularly in low- and middle-income countries (LMICs).

## Articles published in the Health Economics Section over the past 5 years

**Figure 1:**
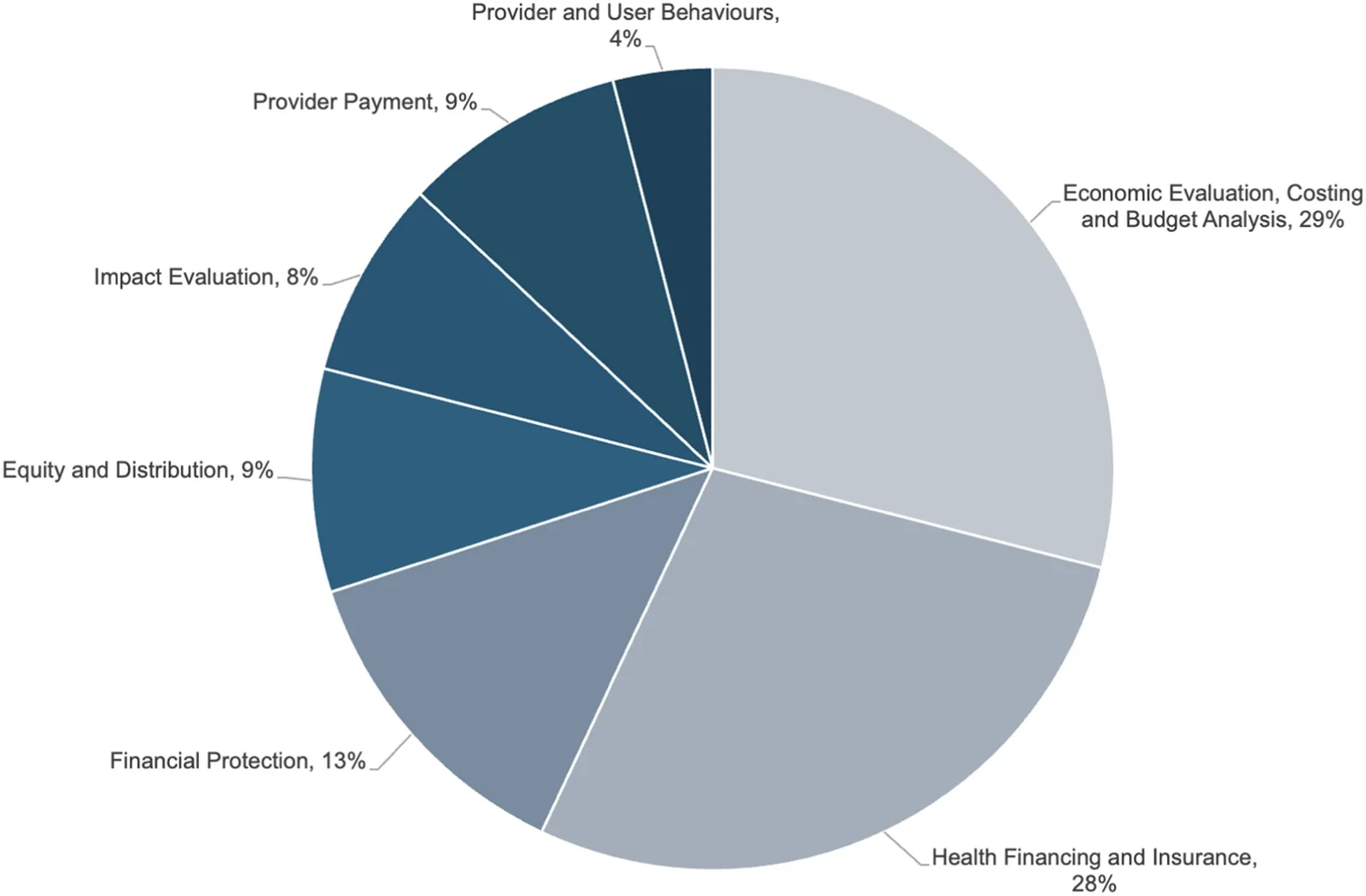
Health economics articles published 2002–2025 by thematic area.

Over the past 5 years (2020–2025), the *Health Policy and Planning* Health Economics Section has published around 220 articles, reflecting a diverse and evolving research agenda in applied health economics and health financing. A review of these publications shows that the journal continues to serve as a key platform for advancing empirical and policy-relevant economic analyses in global health, while also responding to emerging health financing priorities (Fig. 1).

Economic evaluation, costing and budget analysis was the largest cluster, accounting for 29% of articles published over the 5-year period. These articles reported on cost-effectiveness of programmes, interventions, and health services to support planning, budgeting, and priority setting, reflecting the growing demand for evidence on value for money in the context of severely constrained health budgets.

Health financing and insurance represented the second largest cluster (28% of articles), encompassing studies on insurance coverage, pooling arrangements, social protection, and health expenditure patterns. This concentration of papers reflects a sustained policy interest in advancing universal health coverage through prepayment and risk-pooling reforms in LMICs.

Financial protection and household economic burden articles accounted for 13% of the publications in this section, and tended to examine out-of-pocket spending and the measurement of financial risk protection using conventional measures such as catastrophic health expenditure and medical impoverishment at the population-level, as well as in defined subpopulations (e.g. illness-specific and low income). Fewer articles examined poverty vulnerability and the impacts of social protection including cash transfer programs.

Provider payment and equity analyses each accounted for 9% of articles. Provider payment articles explored outcomes of performance-based financing models for different types of providers and in different clinical settings, as well as efficiency outcomes of different payment mechanisms such as capitation and fee-for-service models. The equity analyses examined inequality in access, costs, and outcomes, including across population distributions, as well as fairness in health financing and service utilization.

Impact evaluations represented 8% of articles, including causal evaluations and a small minority of published articles (4%) that examined how health system arrangements shape and impact supply and demand for health care services in LMICs.

## Distribution of articles by manuscript types

**Figure 2:**
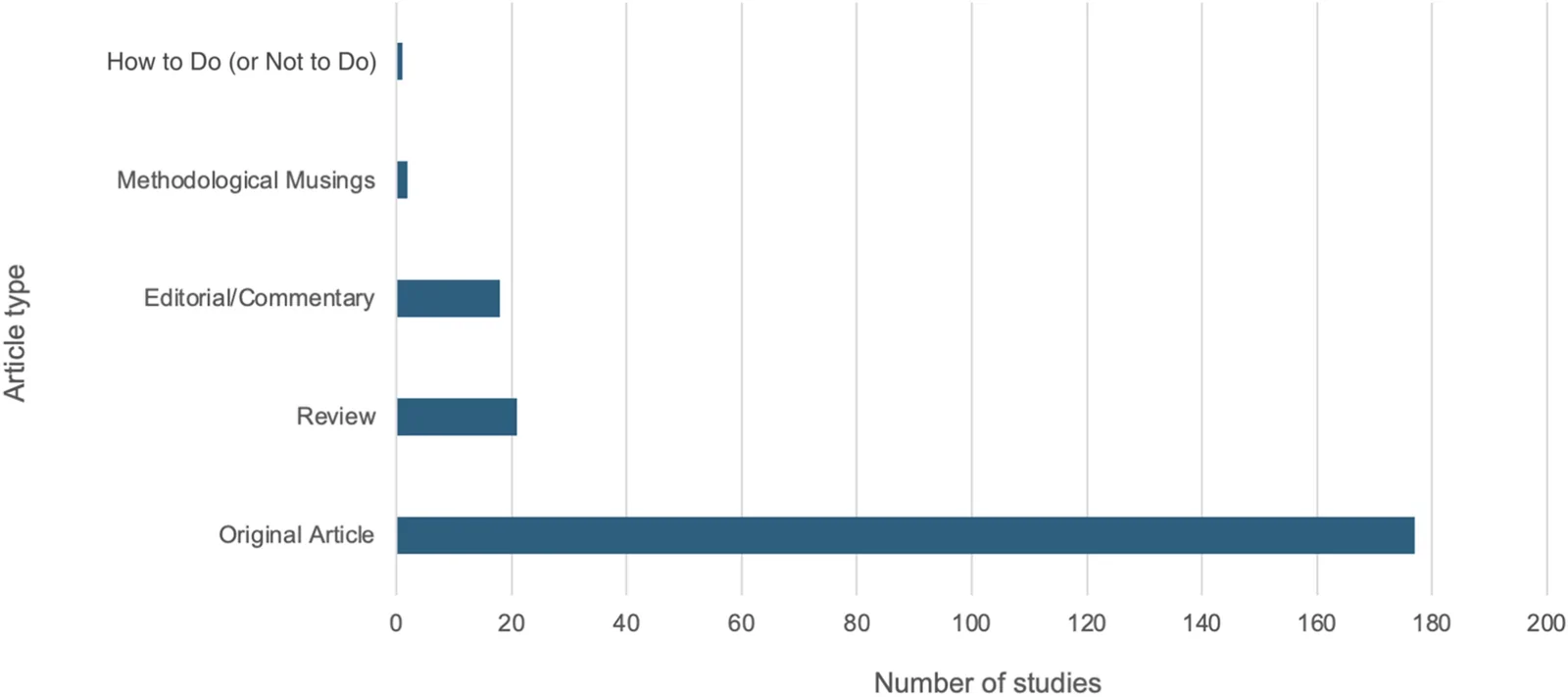
Health economics articles by manuscript type, 2020–2025.

The Health Economics Section was dominated by empirical research, with original research articles accounting for more than 80% of all publications (Fig. 2). This demonstrates the journal’s strong focus on generating new evidence and advancing analytical work. Review articles, commentaries and editorials form a smaller but important share, indicating continued engagement in evidence synthesis and scholarly debate. Only a handful of articles fall into other categories such as Methodological Musings, and How to Do (or Not to Do) articles, yet these include some of the journals most highly cited publications.

## Distribution of articles by publication year

**Figure 3:**
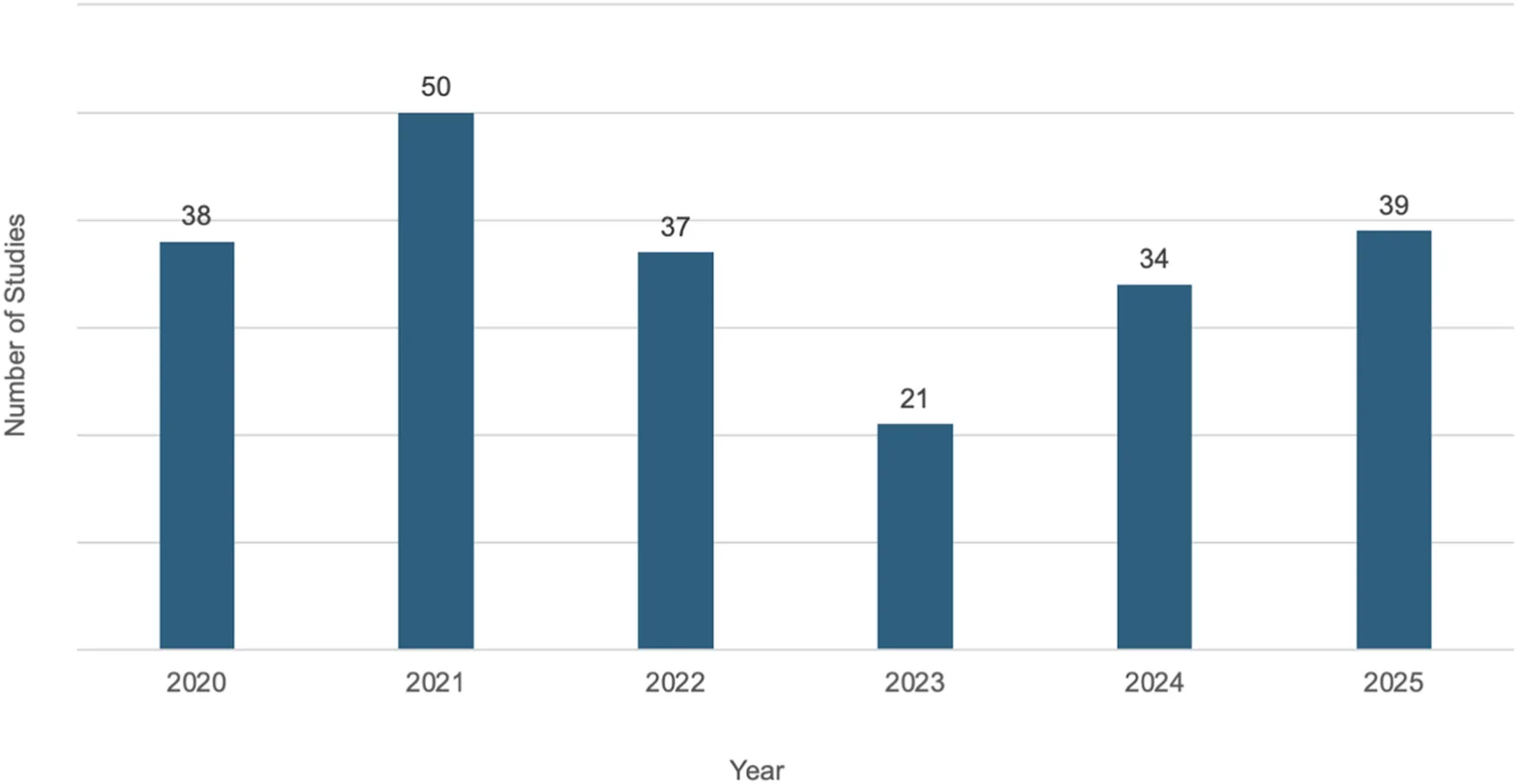
Health economics articles by publication year, 2020–2025.

There is notable fluctuation in the number of health economics articles published each year (Fig. 3). The journal saw its peak in this section in 2021 (*n* = 50 articles), which aligns with the global increase in health-related research activity during the COVID-19 pandemic, as many researchers rapidly generated evidence on health system performance, financing, and policy responses to the pandemic. The notable drop in article numbers in subsequent years likely reflects the postpandemic normalization in research output, shifting funding priorities, or delays in research cycles following disruptions to fieldwork and data collection during 2020–2021. There has been a steady increase in publications since 2023, with 39 papers published in 2025.

## Distribution of articles by geographical regions

**Figure 4:**
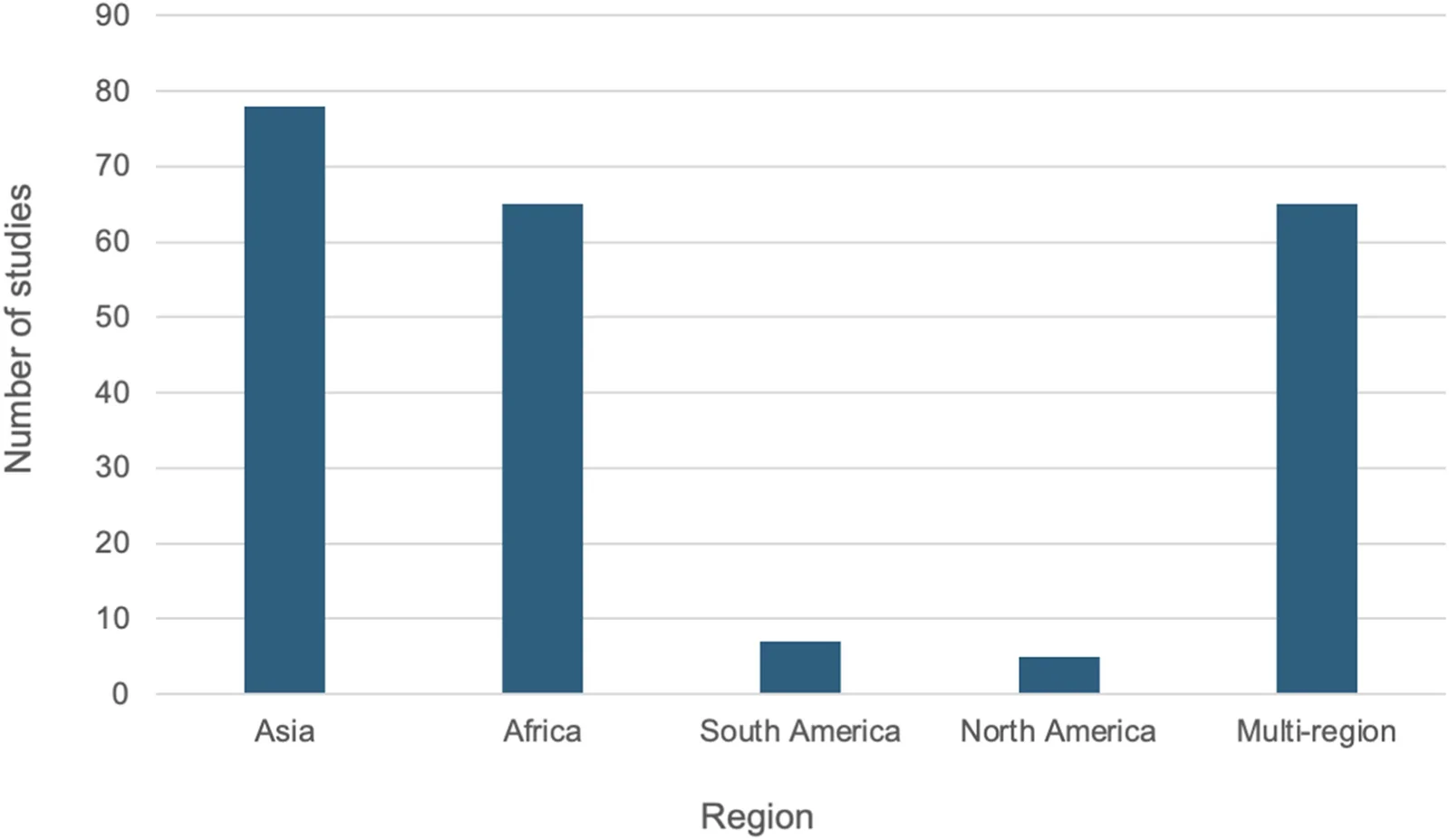
Health economics articles by geographical region.

The geographic distribution of published articles reveals a concentration of articles that originated from Asia and Africa (Fig. 4) with fewer contributions from South America and North America. The concentration of published articles from Asia and Africa may reflect, in part, the journal's sustained focus on health financing challenges in high-burden, resource-constrained settings across these regions. But there has also been a notable growth of local health economics research capacity and expertise across these regions, with increasing numbers of institutions, training programmes, and research networks that are producing local, policy-relevant health economics evidence to guide decision making. Furthermore, in 2023 *Health Policy and Planning* introduced an editorial policy requiring that authorship of submitted manuscripts reflect genuine country-level expertise in the settings studied. It will be of interest to monitor how this requirement is associated (or not) with shifts in the regional distribution of the published research in this section in subsequent years.

Multiregion studies, those drawing on data or comparisons across more than one region, also represented a substantial share of the published articles reflecting comparative and global analyses.

## Advancing health economics: emerging themes, unmet needs, and future directions for *Health Policy and Planning*

While the section’s portfolio is broad and policy-engaged, several areas remain underexplored and warrant greater attention in the journal to meet the evolving needs of health systems in LMICs and beyond.

One notable gap is the limited attention given to fiscal space, taxation, and the long-term sustainability of health financing, including disinvestment strategies and models of self-sustainability in health financing in LMICs. With ongoing global discussions on domestic resource mobilization, there is a clear need for research exploring innovative financing mechanisms and strategies for ensuring fiscal resilience over time. Similarly, the role of the private sector and public–private partnerships has received limited coverage, despite their growing importance in healthcare delivery and financing. Greater engagement with these themes would provide valuable insights into how mixed health systems can be effectively governed and financed.

A further gap in the literature concerns the financing of noncommunicable diseases (NCDs). While the review did not find evidence that infectious disease financing, including pandemic preparedness and response, dominated the published literature, NCD financing was notably underrepresented relative to the scale and trajectory of the NCD burden globally. While financing arrangements that support continuity of care, integrated service delivery, and long-term budget sustainability are health system properties that matter across all conditions, NCD financing warrants specific attention given the particular demands NCDs place on health systems. These demands include, sustained prevention, long-term treatment, multimorbidity management, and cross-sectoral coordination, which expose weaknesses in financing architectures historically designed around episodic, acute care. A further potentially consequential frontier in this space is research on integrated funding models that align financial incentives across health and social care systems to support continuity and coordination.

Emerging priorities such as the economic impact of climate change on health systems and adaptation strategies also warrant greater exploration. Likewise, the expansion of digital health technologies raises critical questions around efficiency, scale-up, opportunity costs, and value for money, yet rigorous economic evaluations and system-level analyses remain limited, especially in LMICs. Mental health economics is another domain with limited representation in *Health Policy and Planning*, despite the substantial burden of mental health conditions and their spillover effects on labor markets, social protection systems, and overall economic productivity. There is a need for stronger economic evidence on both the costs of inaction and the cost-effectiveness of alternative delivery and financing models. Complementing these system-level challenges is a broader need for more work on provider and patient behavior, as behavioral responses to incentives, information, and institutional arrangements increasingly shape the performance of health financing and service delivery reforms.

Methodological innovation represents another important opportunity for the journal. Papers introducing novel approaches, clear methodological guidance, and critical reflection in health financing analysis and economic evaluation are of high relevance to real-world policy development. Of particular current interest are methodological advances in areas such as willingness-to-pay thresholds in LMICs, where rapid economic development and shifting health system priorities are creating demand for context-specific and regularly updated approaches to value assessment.

Likewise, methodological advances focused on embedding equity explicitly across the disciplinary scope of health economics are needed. Approaches that enable intersectional and distributional analyses can make visible how the costs, benefits, and financial risks of health interventions and financing arrangements are experienced differently across gender, income, race, geography, disability, and other intersecting axes of identity. Advancing these methods is essential if the field is to generate evidence that is not only technically rigorous but genuinely responsive to the equity challenges health systems face. The Methodological Musings and How to Do (or Not to Do) paper types in *Health Policy and Planning* provide important opportunities for methodological reflection and for sharing best practices and are typically highly cited manuscripts in the journal.

Finally, as mentioned previously, recent publication patterns show most *Health Policy and Planning* articles within the Health Economics Section mostly reflect research from Africa and Asia, with relatively limited representation from other regions such as South America. Through this editorial, researchers, academics, and practitioners are encouraged to submit high-quality articles that broaden the geographical and thematic diversity of the section.

This reflection back on 5 years of published works in the health economics section at *Health Policy and Planning* highlights a field that is prolific, with geographic variation and methodological diversity. However, there are notable gaps that point to important directions for the journal and the field. As the field continues to advance and local capacity continues to grow across LMICs, there are opportunities for the journal to deepen its engagement with emerging methodological frontiers and to strengthen the diversity of regions, populations, and perspectives represented. The journal is well positioned to lead that agenda.

## Data Availability

Data reported in this manuscript were extracted from the journal's submission records. The underlying data are available from the corresponding author upon reasonable request.

